# Mechanochemical-Aging Synthesis of Bismuth Oxide Nanosheets for Photocatalysis

**DOI:** 10.1021/acsmaterialsau.5c00104

**Published:** 2025-09-19

**Authors:** Delaney J. Hennes, Luke T. Coward, Chase G. Thurman, Oksana Love, Pin Lyu

**Affiliations:** Department of Chemistry and Biochemistry, 8620 University of North Carolina Asheville, 1 University Heights, Asheville, North Carolina 28804, United States

**Keywords:** mechanochemistry, chemical aging, solid-state chemistry, metal oxide nanosheet, photocatalysis, PFOA degradation

## Abstract

Mechanochemistry, old chemistry with new perspectives, has provided unprecedented opportunities for us to pursue a greener and more sustainable future, especially in the exploration of solid-state approaches toward the synthesis of functional nanomaterials. Chemical aging is another environmentally benign and low-energy-demand process that could be controlled precisely with solution environments. In this work, we combined these two approaches to design a mechanochemistry-driven and aging-controlled method for synthesizing nonstoichiometric bismuth oxide nanosheets, which demonstrated a great adsorption capacity and photocatalytic degradation performance toward forever chemicals. With thorough monitoring of the crystal structure and morphological and surface composition changes, the strain-to-defect transformations at the molecular-to-crystal level were proposed to be the dominant growth mechanism. The transient strain accumulation and relaxation from applied mechanical forces during grinding lead to defect-rich metallic bismuth bulk rod structures. The following chemical delamination, achieved through capping ligands and oxygen exposure during aging, produces defect-rich, nonstoichiometric Bi_2_O_2.33_ nanosheet structures. This proof-of-concept synthesis and proposed growth mechanism offer a different perspective toward 2D metal oxide nanosheet design and could help us better design a diverse library of functional nanomaterials through mechanochemistry and chemical aging.

## Graphical abstract

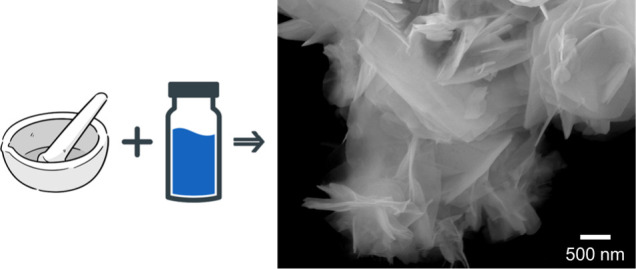

## Introduction

Mechanochemistry, old techniques with new perspectives, has been reawakening the passion for exploring solid-state approaches toward chemical transformations and materials synthesis in a green and sustainable manner, due to its intrinsic solvent-free and eco-friendly nature.^1–8^ More importantly, the driving force of the mechanochemical process, mechanical force, has provided a fundamentally different reaction pathway by promoting intensive, well-oriented, and effective molecular collisions and creating molecular strains and structural defects,^6,9–13^ compared to the conventional thermal or electrostatic driving forces. This perspective has opened new avenues for well-defined nanomaterial synthesis with unique structure–property relationships that the conventional solution-based methods cannot achieve, as well as in a greener way.^4,14–17^ Recent reports have demonstrated great potential in designing metal and metal oxide nanoparticles,^17,18^ nanocomposites,^19^ and hybrid porous materials such as metal–organic frameworks (MOFs) and covalent organic frameworks (COFs) with desired properties.^20–22^

In terms of 2D nanostructure design, two traditional approaches—bottom-up and top-down synthesis—have been widely developed in various synthesis strategies, offering precise control over size, shape, morphology, and dimensions.^23,24^ The formation mechanism behind the scenes can be monitored and reasonably interpreted by advanced in situ spectroscopic^25–27^ or microscopy methods,^28–30^ to identify the critical factors in the structure transformation that lead to property-on-demand synthesis. Specifically, in 2D metal oxide nanosheet synthesis (Scheme 1), chemical vapor deposition, solvothermal method, and chemical exfoliation represent three typical methods. The first two methods start with molecular or atomic precursors for self-assembly growth, while the last method utilizes bulk layered host materials for layer-by-layer delamination. However, these methods rely on sophisticated instrumentation, harsh synthesis conditions, excess solvents, or toxic exfoliating chemicals, which limit their potential scalability.

**Scheme 1:**
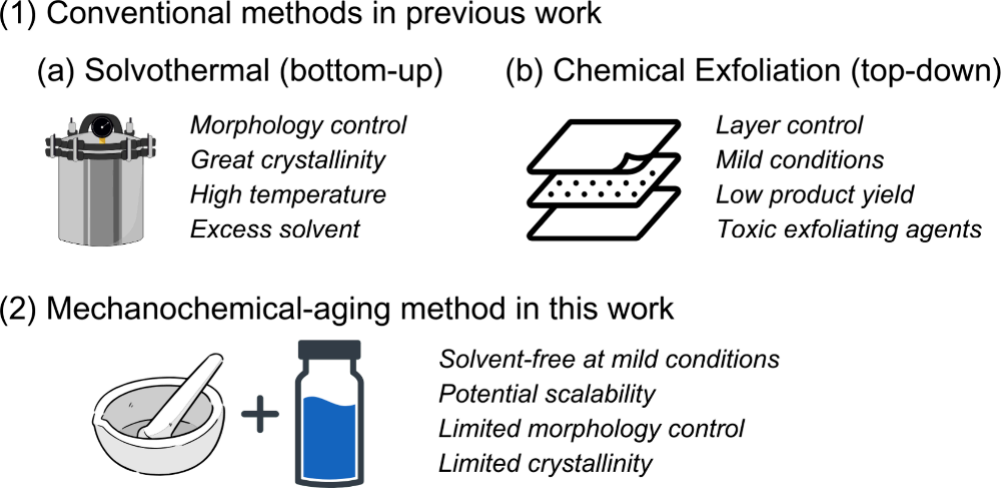
Comparison of Conventional and Mechanochemical-Aging Methods for Bismuth Oxide Nanosheet Synthesis

Mechanochemical synthesis, with its all-solid-state, solvent-free nature, provides an alternative approach. This approach introduces an external applied physical force to initiate intense molecular interactions for mixing and growing (bottom-up) or to spread the shear stress or shock impact uniformly for deformation or delamination (top-down). It has been elegantly demonstrated in various traditional two-dimensional materials syntheses, such as graphene,^31^ MXenes,^32^ and transition-metal dichalcogenides.^33^ Specifically, for bismuth-based materials, nanosheets of bismuthene,^34^ bismuth oxyhalides,^35^ and bismuth nitrates^36^ have been reported previously with the mechanochemical methods. Meanwhile, very few reports are focused on metal oxide 2D nanostructures, probably due to the relatively higher interlayer interactions in those host materials. Thus, a follow-up process is needed to accelerate delamination of the mechanochemically produced bulk materials into the desired 2D nanosheet structure. Recently, Moores’s and Friščić’s groups have successfully demonstrated the combination of mechanochemistry and aging for the extraction and functionalization of biomass derivatives,^37,38^ and functionalized inorganic metal or binary nanoparticle synthesis.^39,40^ Aging, as a spontaneous diffusion-controlled process, can be controlled by the chemical reactivity and solution environment to induce material transformation, further advancing to the targeted structures while minimizing reagent and energy use.^40^

In this work, we developed a mechanochemical-aging process to synthesize nonstoichiometric bismuth oxide (Bi_2_O_2.33_) nanosheets, which demonstrated excellent performance in the adsorption and photodegradation of perfluorooctanoic acid (PFOA). Through the observation of morphological, crystal structure, and surface chemistry changes during the aging process, we propose a strain-to-defect growing mechanism. Mechanical force exerted on metal precursors and reducing agents during solid-state grinding can induce rapid and intense molecular collisions, leading to the generation of strain-rich metallic nanostructures. The following aging process, occurring in an aqueous surfactant-deficient environment, can delaminate the bulk structure into well-defined 2D nanosheets over time with the assistance of ambient oxygen. This approach could potentially be applied to other 2D metal oxide nanostructures.

## Experimental Methods

### Chemicals and Characterizations

All chemicals and reagents were used without any purification. Bismuth­(III) nitrate pentahydrate (99.999%, trace metal basis), PVP (M.W. 40,000), and sodium borohydride (99%, powder) were purchased from Thermo Scientific Chemicals. The morphology and elemental mapping analysis of bismuth oxide nanosheets (Bi_2_O_2.33_ nanosheets) was examined by scanning electron microscopy (SEM, JSM-IT700HR, 15 kV, JEOL) with energy dispersive X-ray spectroscopy (EDS, 0–20 kV energy window, Ultim Max 40: UVA13205 detector, Oxford Instruments). The evolution of the local crystalline structure of Bi_2_O_2.33_ nanosheets was monitored by a powder X-ray diffraction pattern (PXRD, Bruker D2 PHASER Benchtop XRD, Cu tubes, 30 kV, 10 mA) and was processed with DIFFRAC.EVA data analysis software with the Crystallography Open Database (rev.278581). The surface status of functional groups was determined by Fourier transform infrared spectroscopy (FTIR, Shimadzu IRSpirit, 16 scans collected with a resolution of 4 cm^–1^) with the attenuated total reflectance (ATR) mode. The quantification of the PFOA (C_7_F_15_–COOH) and its degradation intermediates, perfluoroheptanoic acid (PFHpA, C_6_F_13_–COOH) and perfluorohexanoic acid (PFHxA, C_5_F_11_–COOH), was determined by liquid chromatography–mass spectrometry (LC-MS, LCMS-8040 Triple Quad LC-MS/MS, Shimadzu Scientific Instruments) in negative electrospray ionization mode. Separation of analytes occurred with the use of a binary solvent gradient between 5 mM ammonium acetate in H_2_O and methanol that ramped from 5% methanol at 0–3 min to 40% methanol from 3 to 16 min and 80% methanol after 16 min. Each liquid was passed through a Phenomenex C18 column (1.8 μm, 100 × 2.1 mm) at a constant oven temperature of 40 °C with a solvent flow rate of 0.25 mL per min. The elution of PFOA occurred at 15.7 min, PFHpA at 13.2 min, and PFHxA at 11.6 min. A ^13^C isotope-labeled PFOA internal standard (Wellington Laboratories) was paired with a ^12^C native PFOA solution to quantify each sample’s concentration. The standard curve for PFOA quantification ranges from 0.5, 1, 5, 10, 25, 50, and 100 ppb, with a limit of detection of 3.6 ppb and a limit of quantification of 11.1 ppb.

### Mechanochemical-Aging Synthesis of Bi_2_O_2.33_ Nanosheets

The mechanochemical grinding process was conducted with an agate pestle and mortar set (Alpha Nanotech, with an inner diameter of 100 mm and an inner depth of 30 mm), and the following aging process was conducted in a 20 mL borosilicate glass vial (VWR scintillation vials) with a polypropylene cap and polyethylene liner. In brief, 1 mmol bismuth­(III) nitrate pentahydrate (Bi­(NO_3_)_3_), 1.975 g polyvinylpyrrolidone (PVP of M.W. 40,000, weight ratio to Bi­(NO_3_)_3_ is about 5:1, serving as the stabilizing capping agents during the nanosheet formation) and 1 mmol sodium borohydride (NaBH_4_) were mixed in dry powder form and grinded for 20 min. It is recommended to handle the grinding of NaBH_4_ under the fume hood to minimize the dust formation or accumulation. The powder mixture turned from white to gray to dark gray gradually (see digital images at different time intervals in Figure 1). Then, the well-ground powder mixture was washed with a 0.5 mM PVP aqueous solution three times. The washing procedure was followed by dispersing the powder mixture in 0.5 mM PVP solutions, centrifuging at 7000 rpm for 10 min, discarding the supernatant solution, and resuspending it in 0.5 mM PVP solutions (through manual shaking). After washing, the powder mixture was dispersed in 0.5 mM PVP solutions in the capped glass vials for the aging process over 10 days. The Day 1 sample referred to the freshly ground powder, and the Days 4, 7, and 10 samples referred to aged powders collected after specific time intervals. The solution color gradually changed from gray to light gray and then to white (see digital images at different time intervals in Figure 1). Eventually, the white solution of Bi_2_O_2.33_ nanosheets was centrifuged to discard the supernatant, and then it was dried overnight in an oven at 80 °C. The whiter powder of Bi_2_O_2.33_ nanosheets was used for further characterization and photocatalysis.

**Figure 1:**
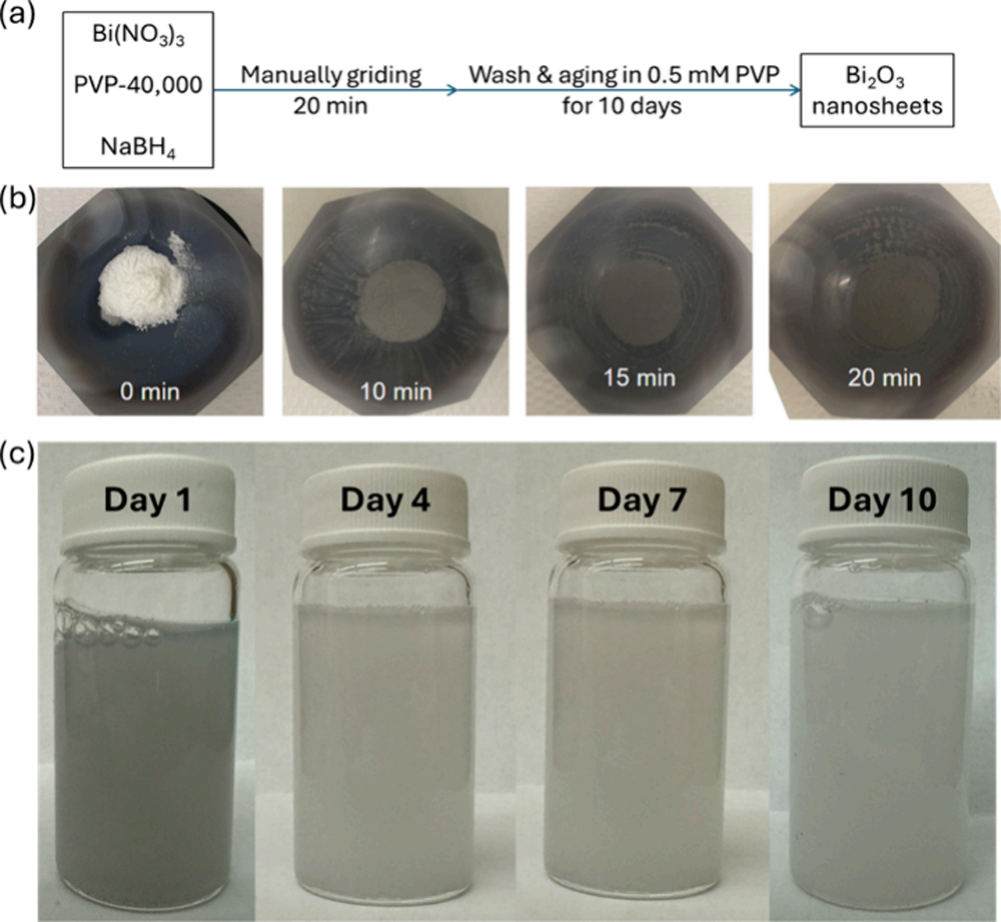
Mechanochemical-aging synthesis protocol and digital images of each step for bismuth oxide nanosheets. (a) Overall synthesis steps of the mechanochemical-aging process. (b) Digital images of the ground samples at different time intervals (0, 10, 15, and 20 min). (c) Digital images of the aging samples in glass vials at different time intervals (1, 4, 7, and 10 days).

### Photodegradation of PFOA by Bi_2_O_2.33_ Nanosheets

The PFOA solution was prepared by serial dilution of the solid compound in a solvent mixture of 80% water and 20% acetonitrile. In a typical photocatalytic reaction, 1 mg of Bi_2_O_2.33_ nanosheet photocatalysts was mixed with 15 mL of 50 ppb PFOA solution in a quartz reaction tube (with a diameter of 15 mm). The reaction mixture was then sonicated for 1 min before being stored in the fridge for 16 h to account for the adsorption–desorption equilibrium. Following a 1-min sonication, the first sample was collected and labeled as the 0-min time point. The reaction quartz tube was then put into a photoreactor (Rayonet RPR-100 Photochemical Reactor) with six ultraviolet lamps (center wavelength of 254 nm, approximately 12.8 mW/cm^2^ at the center) surrounding the quartz tube. All photoreactions were conducted under cooling fans to maintain a stable room temperature. The degradation process was monitored by taking 1 mL solution samples every 30 min for 2 h and centrifuging at 14,000 rpm for 15 min to remove the nanoparticles. The supernatant solutions were filtered by 0.45 μm PTFE syringe filters and saved for LC-MS analysis with an internal standard, ^13^C isotope-labeled PFOA. Typically, 390 μL sample solutions with 10 μL internal standard were mixed in the LC-MS vials and sonicated for 5 min before being put into the autosampler for analysis.

### Photodegradation of PFOA by Anatase TiO_2_ Nanoparticles

The procedures were the same as above, except that 6 mg of anatase TiO_2_ nanoparticles were used as the photocatalysts.

## Results and Discussion

For the synthesis of bismuth oxide nanosheets, the reported traditional methods include solvothermal methods with bismuth molecular precursors and alcohol/polymer surfactants,^41–44^ electrolytic corrosion of metallic Bi,^45^ chemical exfoliation from bulk bismuth oxides,^45–47^ or laser ablation synthesis.^48^ As mentioned above, these methods rely on high energy input (heat, sonication, or laser) or excess exfoliating agents (or solvents), which are neither sustainable nor scalable. Here, we report a mechanochemistry-driven aging approach utilizing limited aqueous surfactant solutions (in principle, recyclable) to synthesize nonstoichiometric bismuth oxide nanosheets, as shown in Figure 1a. In short, an agate pestle and mortar were used to create a mechanochemical environment, in which metal precursors (Bi­(NO_3_)_3_), capping agents (PVP, with a molecular weight of 40k), and reducing agents (NaBH_4_) were manually ground for 20 min. The powder color changes from white to dark gray during grinding (Figure 1b), indicating the transformation from the ionic to metallic states of bismuth. Then, the dark gray powders were washed with 0.5 mM PVP solutions to remove excess reducing agents and polymers. The concentration of 0.5 mM was chosen as it is below its critical micelle concentration (1 mM) to avoid any interference from surfactant self-assembly.^49^ Then, the powders were dispersed in 0.5 mM PVP solutions in capped glass vials for the aging process. The solution color changes from dark gray to white (Figure 1c), indicating the oxidative delamination to form the bismuth oxide nanosheets. Eventually, the aged solution was centrifuged, and white powders of bismuth oxide nanosheets were collected for further characterization and photocatalysis.

To pinpoint the growing mechanism behind the mechanochemical-aging process, morphological, crystal structure, and surface chemistry changes are monitored by using SEM, XRD, and FTIR, respectively. First, after freshly grinding, the gray powder was sent for XRD testing, as shown in Figure 2a as Sample (Day 1). All of the observed diffraction peaks match well with the standard PDF card of metallic bismuth, indicating the successful reduction of molecular bismuth precursors. There is a small peak around 12 degrees, which matches one of the broad peaks of PVP powder (Figure S1), which is anticipated at this stage since the PVP used in the precursor mixture is 5 times the weight of the bismuth salts and has not been washed away. There is another small peak around 29° that matches nonstoichiometric bismuth oxide, which will be discussed below. According to the previous report,^50^ the overall hexagonal crystal structure with a rhombohedral unit cell (as shown by the gray connections in Figure 2b) is commonly observed at ambient conditions for metallic bismuth. Two bilayers within the hexagonal structure are separated by relatively weak van der Waals-like interactions, which provide space and relatively low energy barriers for the following aging process. As follows, during the aging process, with the assistance of PVP surfactant solutions and dissolved ambient oxygen, the metallic Bi gradually oxidizes, and the crystal structure becomes more amorphous, as observed with 3 broad peaks of XRD patterns in Day 4’s and Day 7’s samples. The PVP peaks are not observed in these samples in the XRD, possibly due to the disruptions of the ordered structure in the powder form when they are dispersed in a low concentration solution (0.5 mM). Eventually, on Day 10, a fully oxidized sample is formed, with a crystal structure matching the nonstoichiometric Bi_2_O_2.33_ standard card, and no distinct peaks are observed for metallic Bi anymore. Additionally, Figure S2 compares standard patterns for bismuth subcarbonate and three distinct phases of stoichiometric bismuth oxide with those of the Day 10 sample. None of these reference patterns match the observed pattern of the Day 10 sample, ruling out the presence of these phase impurities in our material. This mixed-valent (Bi^2+^ and Bi^3+^) electron-rich structure of nonstoichiometric Bi_2_O_2.33_ was previously reported in research using traditional synthesis methods mentioned above, which demonstrated excellent properties, such as UV-emitting photoluminescence, supercapacitance, ferromagnetism, and photocatalysis.^41,43,47^ To the best of our knowledge, there have been no reports on the mechanochemistry-driven synthesis of this unique structure. To be specific, as shown in Figure 2c, the overall tetragonal structure consists of a mixture of Bi_2_O_3_ and Bi_2_O_2_ layers within the lattice, possibly coming from the oxidation of metallic bismuth in the original bilayer structures before aging. This unique structure, featuring the stereochemically active lone pair of Bi^3+^ and the electrostatically electron-rich site of Bi^2+^, could potentially contribute to interlayer flexibility and defect formation, ultimately leading to the final nanosheet structure. This has been observed in other bismuth-based materials with preferred layered structures as well.^51–53^

**Figure 2:**
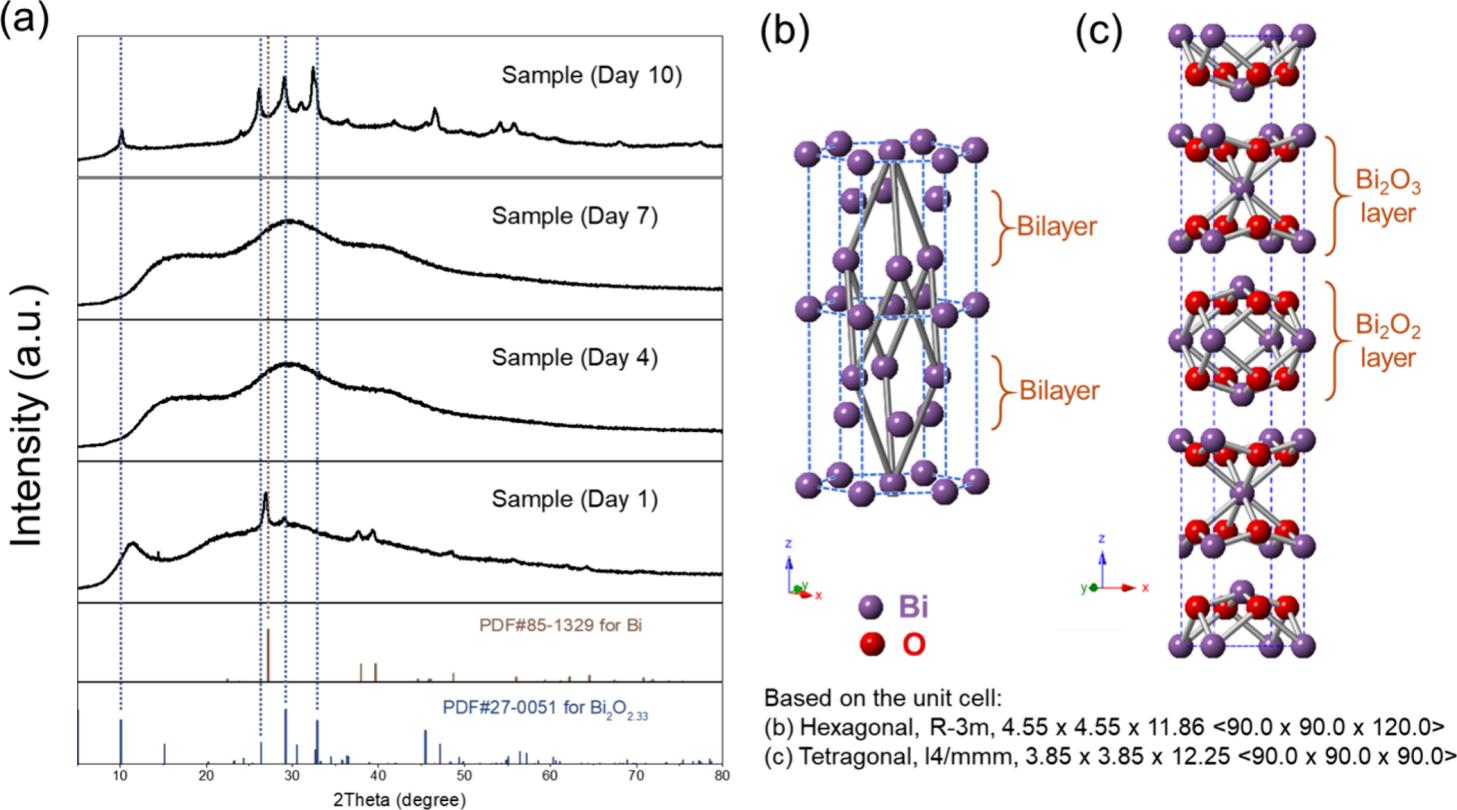
Structural evolution for the mechanochemical-aging synthesis process. (a) XRD spectra of samples collected on Day 1 (freshly made) and Days 4, 7, and 10 with the standard PDF reference cards for metallic Bi and nonstoichiometric Bi_2_O_2.33_. Schematic presentation of the crystal structures of (b) metallic Bi and (c) nonstoichiometric Bi_2_O_2.33_.

Moving forward, the SEM images of freshly made (Day 1) samples and samples at different intervals during the aging process were collected for tracking morphological changes, along with EDS mapping to confirm the elemental composition and compare the spatial distribution of these elements. As shown in Figure 3, the bulk rod microstructure of metallic Bi after grinding and washing on Day 1 was observed with a relatively small amount of well-defined hexagonal thin nanosheets. These nanosheets have a relatively smooth surface, and the edge length is about 1 μm with a lateral width of 2 μm. The EDS full-range scan results also confirmed the dominant element compositions of carbon, oxygen, and bismuth in Figure S3, in which the carbon comes from both the carbon tape used in the sample preparation and the PVP polymer residues. For the EDS mapping in Figure S4, the distributions of Bi and O are relatively and consistently uniform across the sample, while the oxygen amount is significantly higher than the bismuth (estimated based on the spectra intensity counts and relative atomic mass). It is possible due to the residual PVP polymer still adsorbed on the surface, and is ready to participate in the next delamination process. This is also observed in the FTIR spectrum of O–H bending, C–H bending, and C–N stretching, possibly from the PVP, as shown in Figure S5, compared to the FTIR spectrum of PVP powder. It is noteworthy that very weak Bi–O bonding and a hydrophobic surface with almost no O–H stretching are also observed in Day 1’s sample, combined with the evidence of PVP, dominant metallic Bi peaks, and a small peak of Bi_2_O_2.33_ from the XRD analysis above. It is suggested that after freshly grinding, the metallic Bi bulk microrods formed as the aging precursor for the subsequent delamination process, and small amounts of oxide nanosheet structure began to form adjacent to the bulk rod, indicating a delamination pathway rather than nucleation from the aging solution.

**Figure 3:**
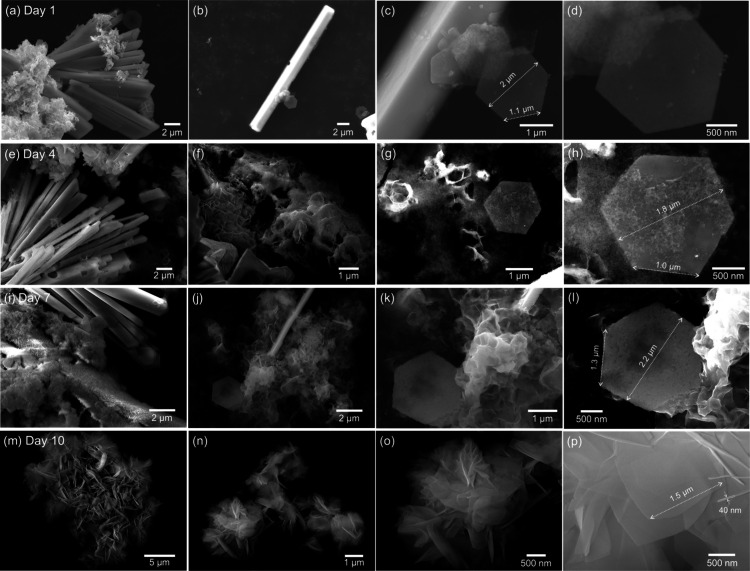
Morphology evolution for the mechanochemical-aging synthesis process. SEM images of samples collected on (a–d) Day 1 (freshly made), (e–h) Day 4, (i–l) Day 7, and (m–p) Day 10.

After being ground and washed, the samples were dispersed in the 0.5 mM PVP solution in a capped glass vial for the aging process. As shown in the Days 4 and 7 samples in Figure 3, the bulk metallic Bi microrods started to peel off into the nanosheet structures with amorphous transition intermediates on the sheet surface, under an ambient oxygen environment. Specifically, on Day 4, much smaller rods were observed, and a larger amount of amorphous sheet-like structures formed either separately or on the well-defined hexagonal nanosheet surface. A significant amount of PVP signals, along with Bi–O and Bi–O–Bi bonds, were observed in the FTIR spectra (Figure S5). On Day 7, the rod’s surface started to form more amorphous, sheet-like structures, suggesting a further delamination process with the assistance of PVP. The individual nanosheet began to separate from the bulk structure, revealing a much cleaner surface, possibly due to the repulsion effect of the PVP polymer fully inserted between the bilayer structures of the metallic bismuth. Overall, the nanosheet dimensions of these nanosheets remain relatively consistent throughout the aging process, suggesting that the benefits of controlled chemical environments can be further regulated to adjust these dimensions to the targeted ones. It is noted that in a separate experiment (not shown here), we sealed the aging glass vials with parafilm to limit oxygen exchange, and the aging process was significantly slowed down, suggesting the necessary contribution of both PVP and oxygen. We are currently tracking the amounts of PVP and the amount of oxygen involved in the process to explore a more diverse library of nanosheet structures. Additionally, exposure to light or heat and bubbling with air into the system could potentially accelerate the aging process, which will be addressed in more detail in our future work.

By Day 10, the delamination was complete with stacked thin nanosheets, and no rods or amorphous structures remained; this chemical transformation matched the color change observed above (from light gray to white). These nanosheets are about 1.5 μm in edge length and about 40 nm in thickness, as shown in Figure 3, which are a little larger than the starting hexagonal sheets. The clearly separated C–H bending and C–N stretching from the residual PVP polymer are observed in Figure S5, along with significantly stronger Bi–O–Bi and Bi–O bonding signals. It is possible that these polymeric capping ligands are more oriented on the nanosheet surface, preventing the materials from further overgrowth into larger bulk structures. More interestingly, changes to the hydrophilic surface with clear O–H stretching and O–H bending being observed are possibly due to the electron-rich defect sites coming from the nonstoichiometric Bi_2_O_2.33_ structures, as confirmed from the XRD above. These defect sites are beneficial for the adsorption and photocatalysis performance, as will be discussed below. The EDS elemental mapping analysis in Figure S4 indicates a much higher percentage of Bi on the sample surface, suggesting a relatively cleaner surface with less PVP or an amorphous structure residing on the surface. This is consistent with the morphological and crystal structure observations above. It is in our current progress to track the tipping point of these sudden changes from amorphous to well-defined oxide nanosheet structure; however, some in situ monitoring, microscopic or spectroscopic techniques,^54^ are necessary to capture that transition, which will be addressed in our future work.

Based on the collective evidence of the crystal structure, morphology, and surface composition observed above, we propose a possible mechanochemical-aging growth mechanism, as shown in Table 1, that features strain-to-defect transformations at the molecular-to-crystal level. As mentioned in the introduction, mechanical energy generated by grinding can be compressively and uniformly dispersed into the precursor reactants, promoting effective molecular collisions and leading to the reduction of ionic salts into metallic Bi. Meanwhile, the transient strain accumulation and relaxation resulting from applied mechanical forces could lead to defect-rich surfaces in the formed metallic Bi bulk structures, which will serve as sites for the formation of metal oxide nanosheets during aging. More importantly, these defects remain in the final nanosheets. More specifically, well-defined defect-rich metallic Bi bulk rods were formed with a hydrophobic surface after freshly grinding on Day 1. The hexagonal crystal structure, featuring bilayers in between, began to delaminate into nanosheets with the assistance of PVP polymer and ambient oxygen, undergoing amorphous transitions over Days 4 and 7. The freestanding PVP polymer also served as a thickness control agent, limiting overgrowth to the bulk structure (C–N stretching evolved over time). Eventually, the aging process was completed, leaving no bulk rods, and nonstoichiometric Bi_2_O_2.33_ 2D nanosheets were formed with a hydrophilic surface and PVP stabilizing agents. These results confirmed the critical contributions of both mechanical force and chemical environment to the mechanochemical-aging synthesis, supporting the interpretation of the proposed mechanism. It is noteworthy that this mechanochemistry-driven and aging-controlled approach could be potentially expanded into other metal oxide 2D nanosheet structure designs, which is in our current progress to tackle.

**Table 1:**
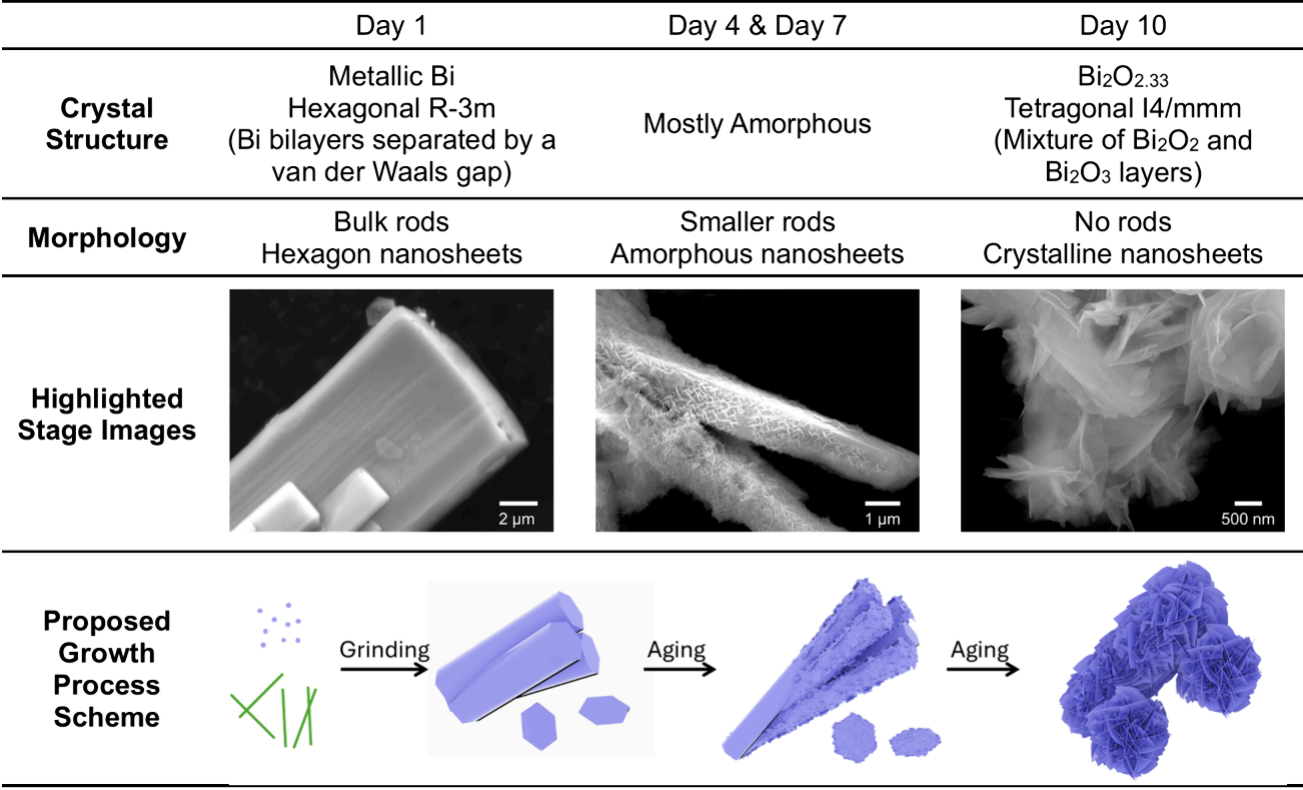
Proposed Mechanochemical-Aging Growth Mechanism and Related Evidence^a^

a Note: The dots in the growth scheme represent the bismuth precursors, and lines represent the PVP polymer.

Finally, the adsorption capacity and photocatalytic degradation of PFOA were used to evaluate the performance of these nanosheet structures, compared to the commercial 15 nm anatase TiO_2_ nanoparticles. PFOA, as one of the two most detected per- and polyfluoroalkyl substances (PFAS) in various environmental compartments,^55^ has been persistently existing and contaminating drinking water. Photocatalytic degradation has provided an alternative approach to tackle these forever chemicals in a greener and more sustainable way.^56^ As shown in Figure S6, with the same amount of photocatalysts (6 mg), Bi_2_O_2.33_ nanosheets demonstrated a much higher adsorption capacity (98%) than the 15 nm TiO_2_ nanoparticles (7%). It is possibly due to the larger surface area and the more electron-rich defect sites of the nanosheet structures. To account for the larger amount of adsorption, 1 mg of Bi_2_O_2.33_ nanosheets was used for the following photocatalytic degradation performance, while 6 mg of 15 nm TiO_2_ nanoparticles was used. Typically, after 16 h of adsorption–desorption equilibrium, the first sample was collected, designated as 0 min in the photocatalysis process. As shown in Figure 4, the Bi_2_O_2.33_ nanosheets demonstrated much faster degradation of the PFOA, almost 80% after 2 h of UV light irradiation, while the 15 nm TiO_2_ nanoparticles showed less than 20% degradation. Comparing the apparent rate constants of these two reactions, the Bi_2_O_2.33_ nanosheets showed about 10 times faster than the TiO_2_ nanoparticles, not to mention that the catalyst amount used was only 1 mg compared to 6 mg. These results demonstrated the great potential of the as-synthesized Bi_2_O_2.33_ nanosheets in terms of adsorption capacity and photocatalytic efficiency. More importantly, the SEM and EDS elemental mapping analysis of the Bi_2_O_2.33_ nanosheets after the photocatalytic reactions, shown in Figure S7, suggests the stability of these nanostructures with no obvious deformation or loss of the bismuth elements, which are critical for recycling and reuse of these photocatalysts. We are currently working on designing a photoreactor that better utilizes such nanosheets, either dispersed or loaded onto supporting materials, for long-term recyclability tests.

**Figure 4:**
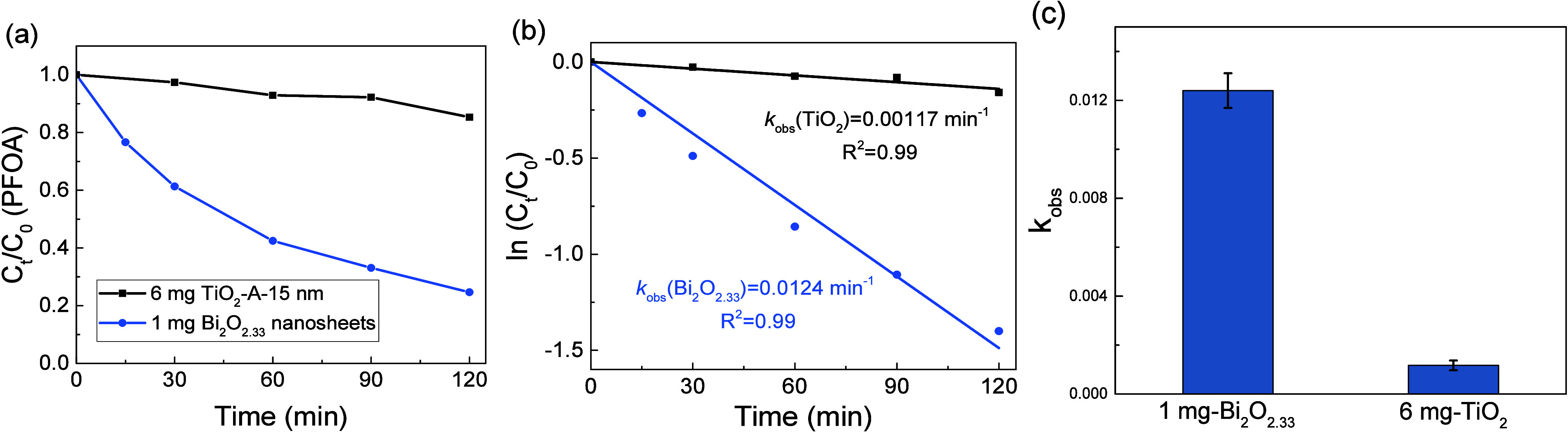
Photocatalytic performance of Bi_2_O_2.33_ nanosheets and anatase TiO_2_ nanoparticles. (a) Degradation percentage after 16 h of adsorption–desorption equilibrium under UV light. (b) Observed apparent rate constants extracted from (a). The linear fits suggest a pseudo-first-order process for the PFOA reactants. (c) Observed rate constants comparison from 3 different replicated experiments. All error bars represent one standard deviation of the mean.

Another critical factor in the photocatalytic PFOA degradation is to confirm the short-chain intermediates formed and potentially establish a balance of carbon and fluorine ions across the degradation process, ultimately targeting nontoxic products. The common degradation pathway of PFOA (C_7_F_15_–COOH) is followed by stepwise defluorination to form intermediates such as perfluoroheptanoic acid (PFHpA, C_6_F_13_–COOH) and perfluorohexanoic acid (PFHxA, C_5_F_11_–COOH). Specifically, in heterogeneous photocatalysis, the electrons generated from photoexcitation can react with adsorbed water to form hydrated electrons or with oxygen to form superoxide radicals to attack PFOA, while on the other hand, the highly oxidizing holes can be either directly involved in the degradation or react with water to form the hydroxyl radicals to attack PFOA.^57^ Either pathway will generate the short-chain byproducts mentioned above. As shown in Figure 5, the C7 and C6 short-chain intermediate byproducts were detected by LC-MS with a relative concentration increase over time, suggesting the successful degradation of PFOA by the Bi_2_O_2.33_ nanosheet photocatalysts, rather than only the adsorption effect. Eventually, the proposed mechanism is illustrated in Figure 5b, which shows contributions from both photoexcited electrons and holes to the degradation pathway. The rich defect sites on the surface of the nonstoichiometric Bi_2_O_2.33_ nanosheet structure could potentially serve as trapping centers, thereby benefiting charge separation of the electron–hole pairs and enhancing the overall photocatalytic performance, as reported in previous work.^58^ In terms of further tracking the destiny of these intermediates and the overall balance of carbon and fluorine ions, it is beyond the scope of this proof-of-concept work to develop a mechanochemistry-driven and aging-controlled synthesis of 2D nanosheet structures.

**Figure 5:**
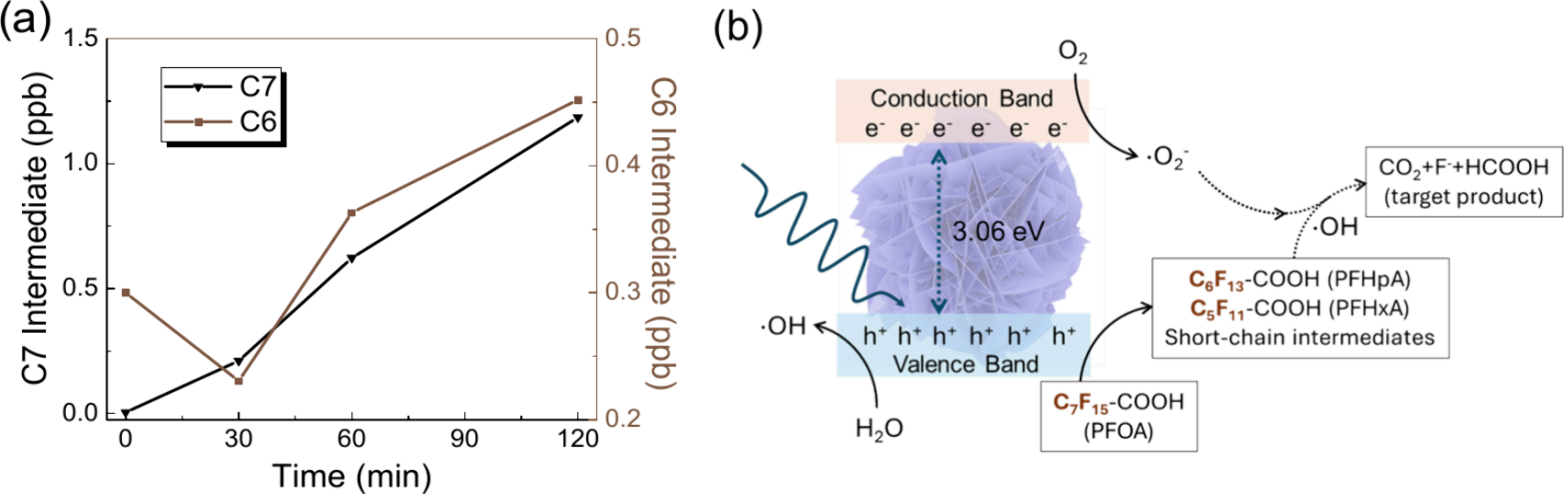
(a) Photocatalytic degradation intermediates detected with the Bi_2_O_2.33_ nanosheet photocatalysts. (b) Proposed photocatalysis mechanism to break down the PFOA, potentially to short-chain or environmentally benign products. C7 represents perfluoroheptanoic acid (PFHpA, C_6_F_13_–COOH) and C6 perfluorohexanoic acid (PFHxA, C_5_F_11_–COOH). The band gap value was retrieved from ref 43.

The reproducibility of the synthesis was confirmed by various characterizations of replicated samples of Days 1, 4, 7, and 10 (Figures S8–S13). More specifically, in the replicated samples, the overall structural evolution (XRD patterns in Figure S8) was the same as that in the original batches, while the overall morphology change (SEM images in Figures S9–S12) during the aging process was similar to that in the original batches. More hexagonal sheets were observed during the aging process in the replicated samples (Figure S10), which may be attributed to some deviations in the grinding process. We are currently working on utilizing a ball mill to more consistently reproduce this process. The surface composition change (FTIR in Figure S13) was observed to have a similar trend in the repeated samples, with more Bi–O and Bi–O–Bi bonds observed during the aging process. There were some deviations from the PVP and O–H signals in the aging samples of Days 4 and 7, probably due to the disordered structure of PVP adsorbed on the amorphous nanostructure surface or in water. We are further tracking that change more quantitatively with more in situ methods, as mentioned earlier. Meanwhile, the Day 1 and Day 10 samples were the same in terms of surface composition, which, in general, should not affect our mechanistic interpretation above.

## Conclusions

In summary, we developed a mechanochemical-aging approach for the synthesis of nonstoichiometric Bi_2_O_2.33_ nanosheets, exhibiting excellent adsorption capacity and photocatalytic degradation performance toward forever chemicals. Different stages of morphological, crystal structure, and surface composition changes were observed and correlated to our proposed growth mechanism. Our mechanistic interpretation focuses on the effects of transient strain accumulation and relaxation resulting from applied mechanical forces during grinding, as well as controllable chemical delamination through capping ligands and oxygen amount during aging. This mechanism features strain-to-defect transformations at the molecular-to-crystal level, which could potentially extend to other 2D metal oxide nanostructure designs.

## Supplementary Material



## Notes

XRD pattern of PVP powder (Figure S1); XRD patterns of Day 10 samples compared to other possible phases (Figure S2); EDS full-range scan spectra of samples from Days 1, 4, 7, and 10 (Figure S3); EDS mapping of Bi and O of samples from Days 1, 4, 7, and 10 (Figure S4); FTIR spectra of samples from Days 1, 4, 7, and 10 (Figure S5); adsorption performance comparison between Bi_2_O_2.33_ nanosheet and TiO_2_ nanoparticles (Figure S6); EDS full-range scan spectrum and mapping of the samples after photocatalyzed reactions (Figure S7); XRD patterns of replicated samples (Figure S8); SEM images of replicated samples from Days 1, 4, 7, and 10 (Figures S9–S12); and FTIR spectra of samples from Days 1, 4, 7, and 10 (Figure S13) (PDF)
