# Supplementary material for: Mechanochemical-Aging Synthesis of Bismuth Oxide Nanosheets for Photocatalysis
Source: ACS Mater Au. 2025 Sep 19;5(6):1009–17. doi: 10.1021/acsmaterialsau.5c00104 (PMC12616431; doi:10.1021/acsmaterialsau.5c00104)
Supplement: Supplementary file 1 [file mg5c00104_si_001.pdf]

Supporting Information
Mechanochemical-Aging Synthesis of Bismuth Oxide Nanosheets for Photocatalysis

Delaney J. Hennes,† Luke T. Coward,† Chase G. Thurman, Oksana Love,* and Pin Lyu*
Department of Chemistry and Biochemistry, University of North Carolina Asheville, 1 University Heights, Asheville, North Carolina 28804, United States.

† These authors contributed equally.

*Corresponding Authors: olove@unca.edu and plyu@unca.edu

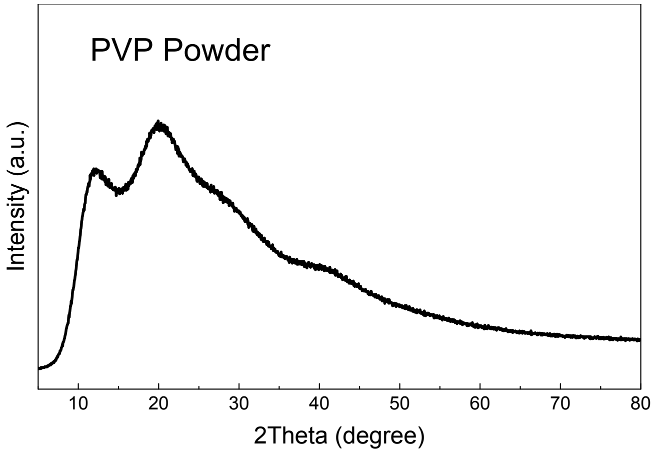

Figure S1. XRD pattern of PVP powder.

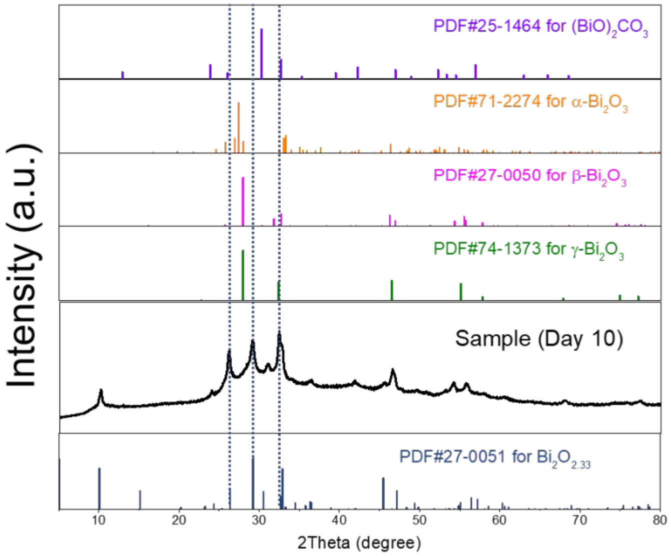

Figure S2. XRD pattern of the synthesized Day 10 sample compared to the standard PDF cards of other possible phases. From top to bottom: bismuth subcarbonate, $(BiO)_2CO_3$ and three different phases of bismuth oxide (α-, β-, and γ- Bi_2O_3).

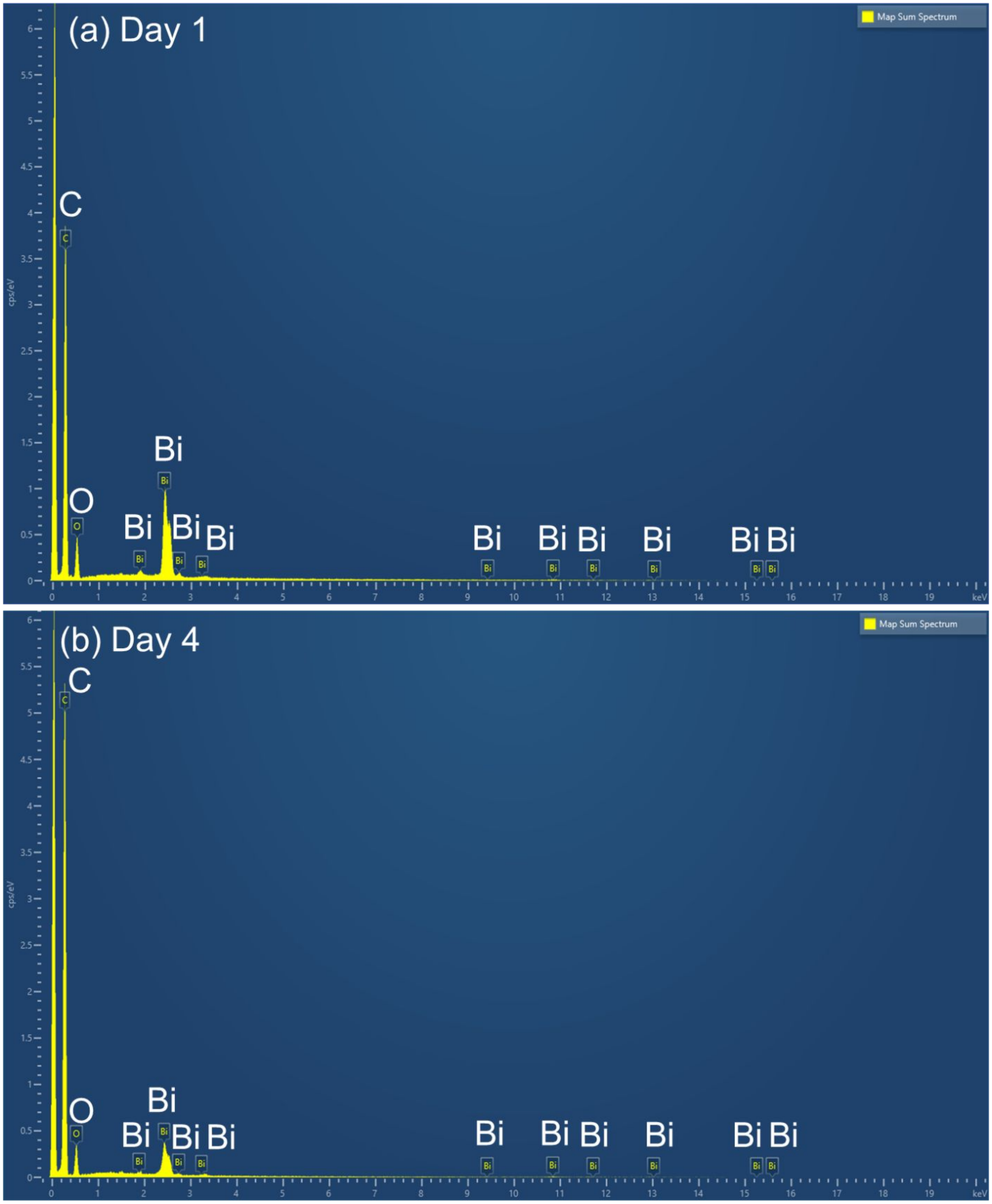
(a) Day 1
Map Sum Spectrum
cps/eV
C
O
Bi
keV
(b) Day 4
Map Sum Spectrum
cps/eV
C
O
Bi
keV

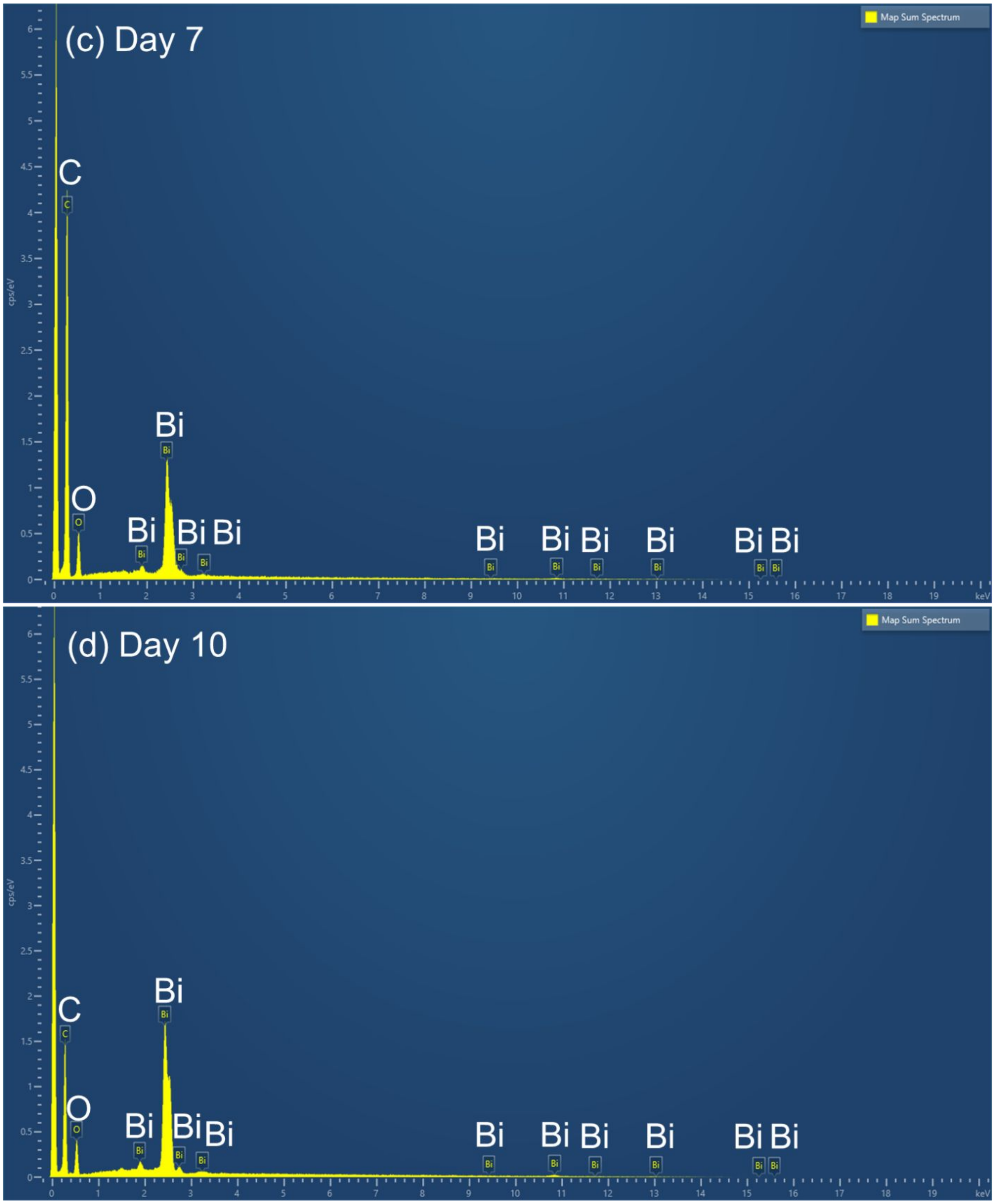

Figure S3. SEM-EDS full-range scan spectra in the samples collected on day 1(freshly-made), day 4, day 7 and day 10.

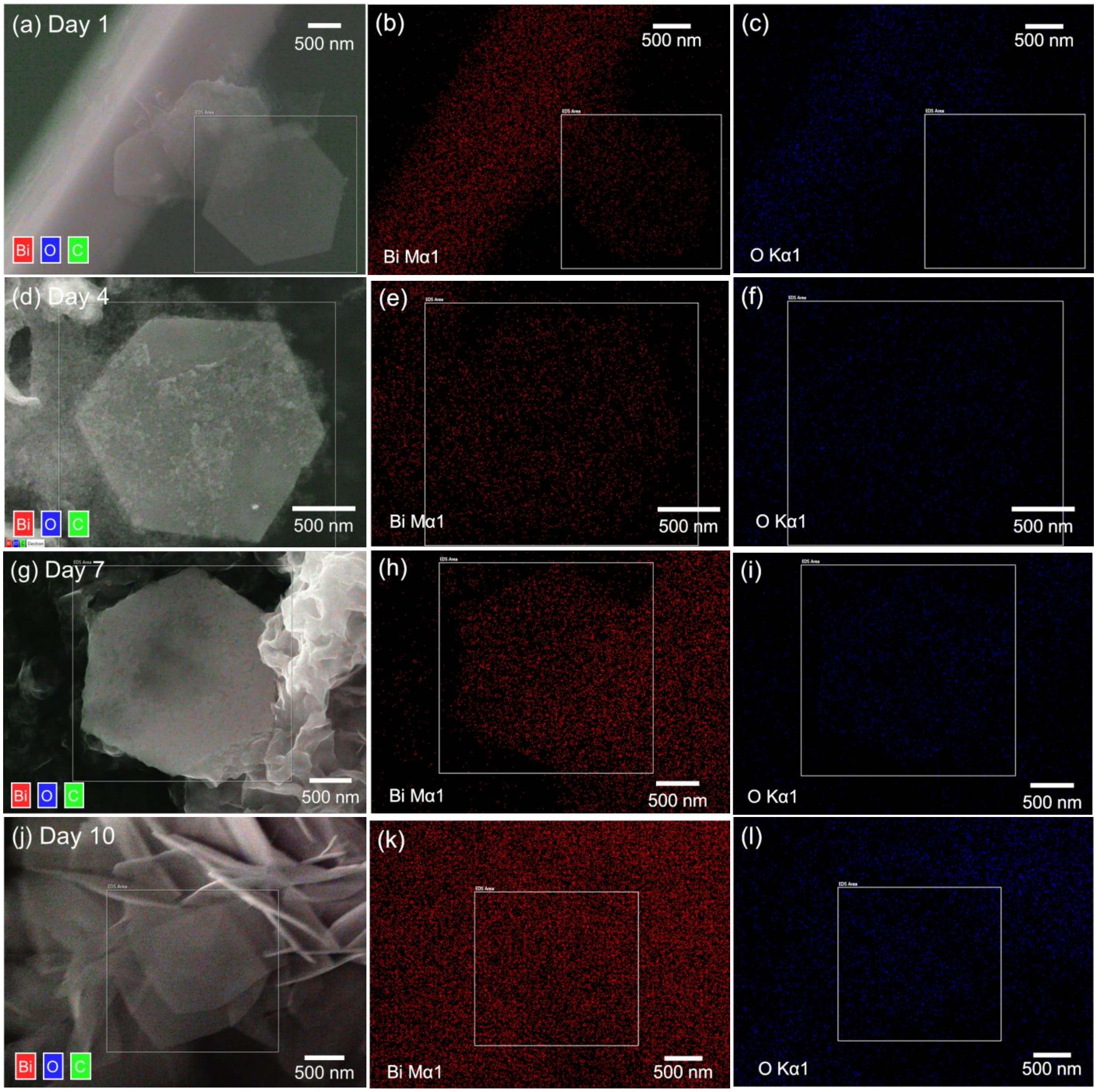

Figure S4. SEM-EDS mapping analysis of samples collected on (a-c) day 1(freshly-made), (d-f) day 4, (g-i) day 7 and (j-l) day 10. Elemental mappings for Bi and O were adjusted with a contrast (-40%) and brightness (+40%) correction to increase their visibility.

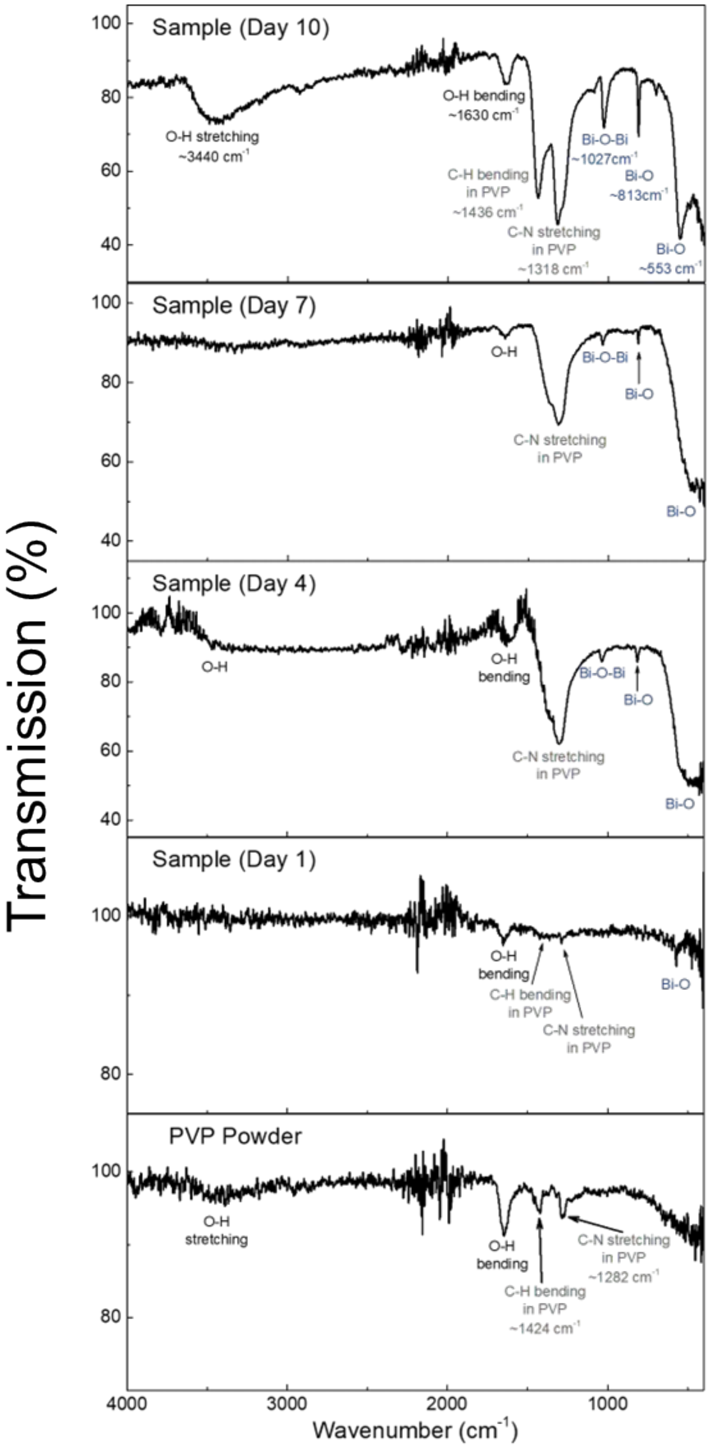

Figure S5. Surface status evolution analysis of samples collected on day 1(freshly-made), day 4, day 7 and day 10.

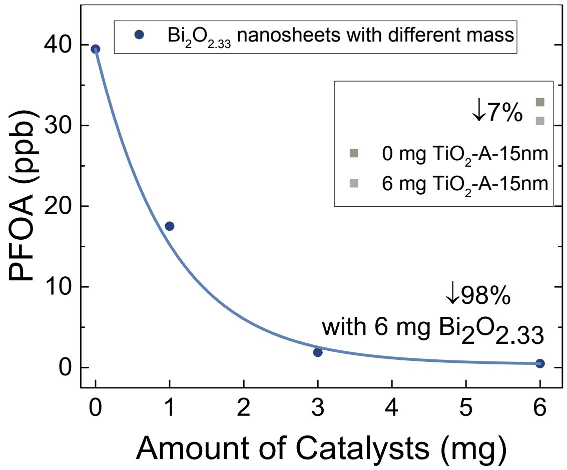

Figure S6. Adsorption performance comparison between $Bi_2O_{2.33}$ nanosheet and TiO_2 nanoparticles. The adsorption experiment was conducted in the same conditions as the photocatalytic degradation experiments described in the Experimental section, except that the light irradiation was not performed before measuring the concentration by LC-MS.

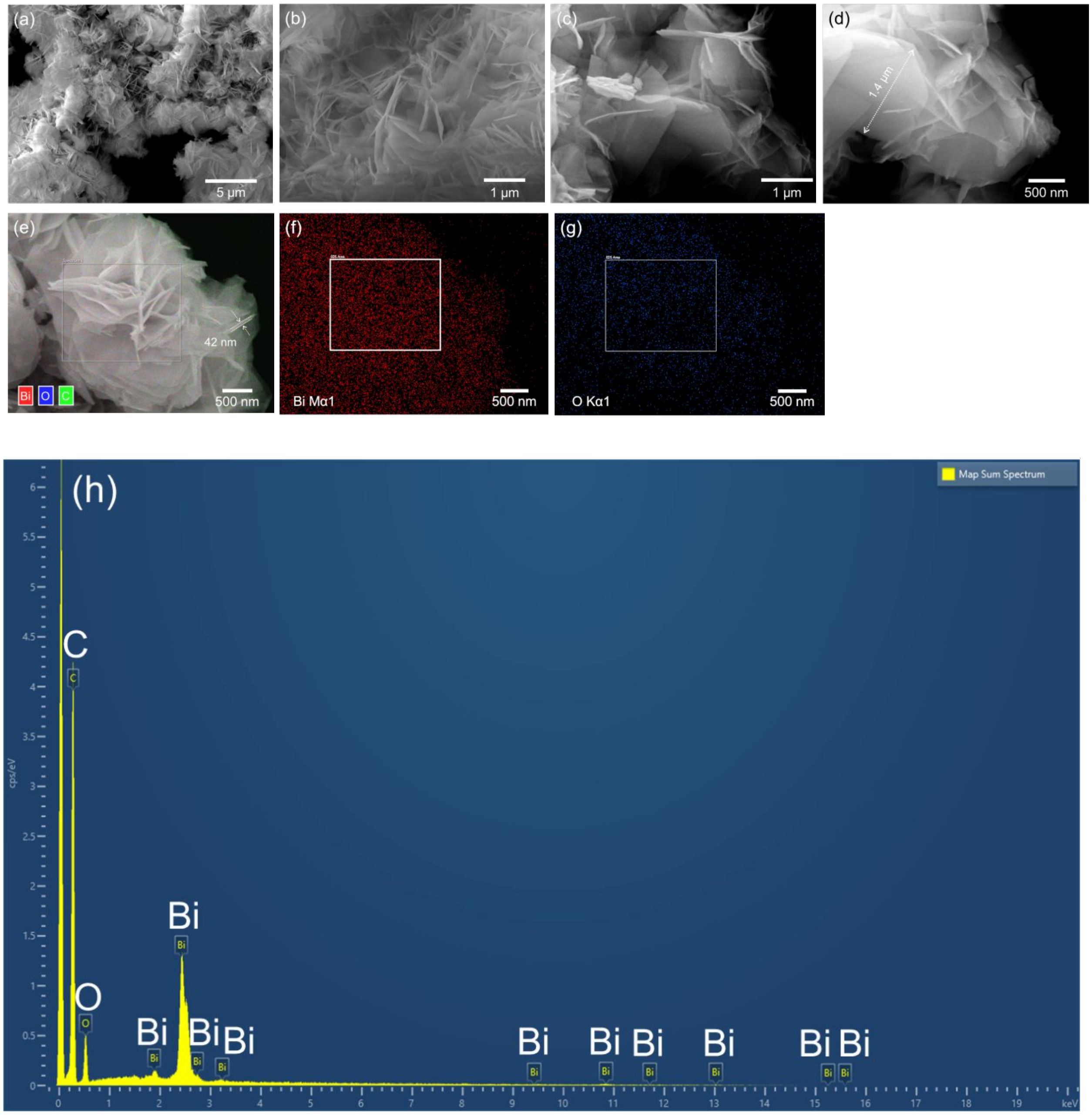

Figure S7. Morphology and elemental analysis after photocatalyzed reactions of $Bi_2O_{2.33}$ nanosheet photocatalysts. SEM images, EDS full-range scan spectrum and elemental mapping analysis. Elemental mappings for Bi and O were adjusted with a contrast (-40%) and brightness (+40%) correction to increase their visibility.

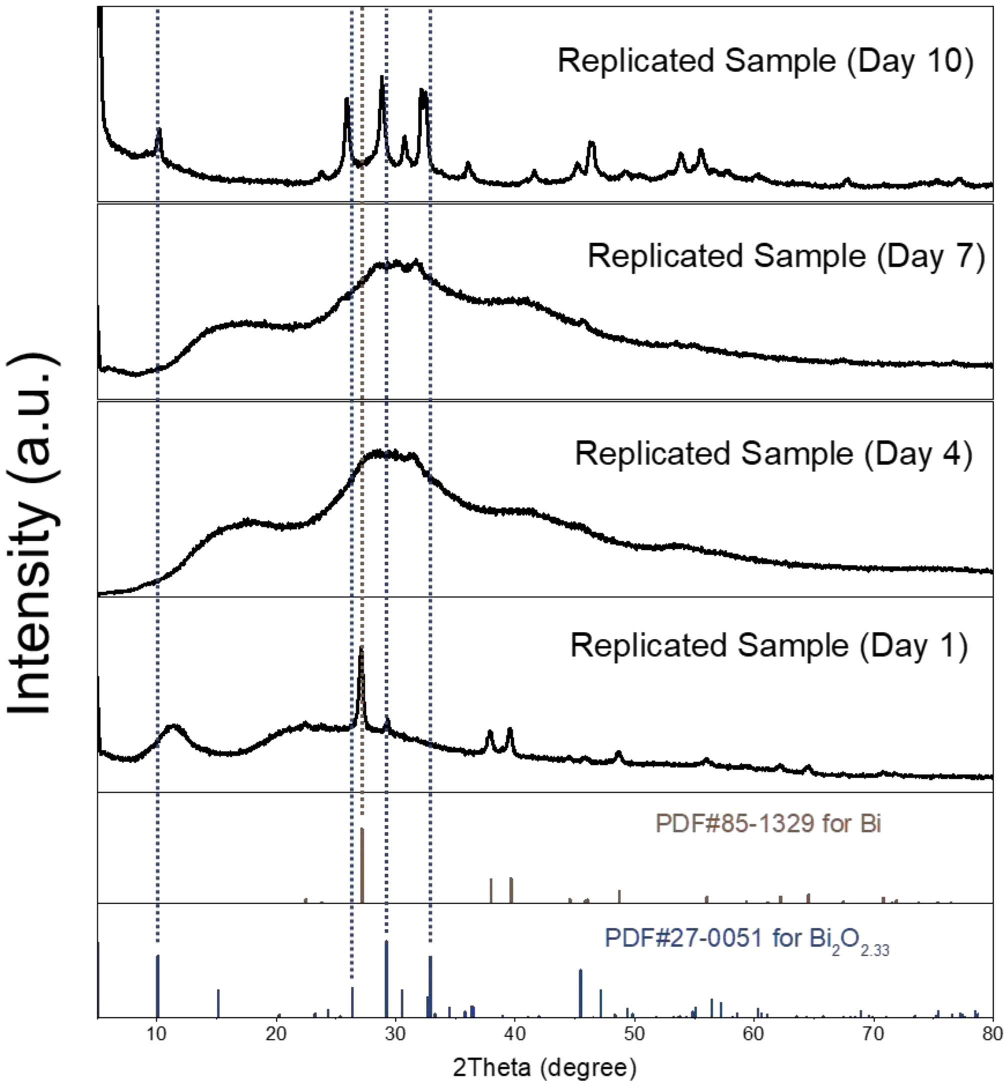

Figure S8. XRD patterns of replicated samples collected on day 1(freshly-made), day 4, day 7 and day 10 with the standard PDF reference cards for metallic Bi and non-stoichiometric $Bi_2O_{2.33}$.

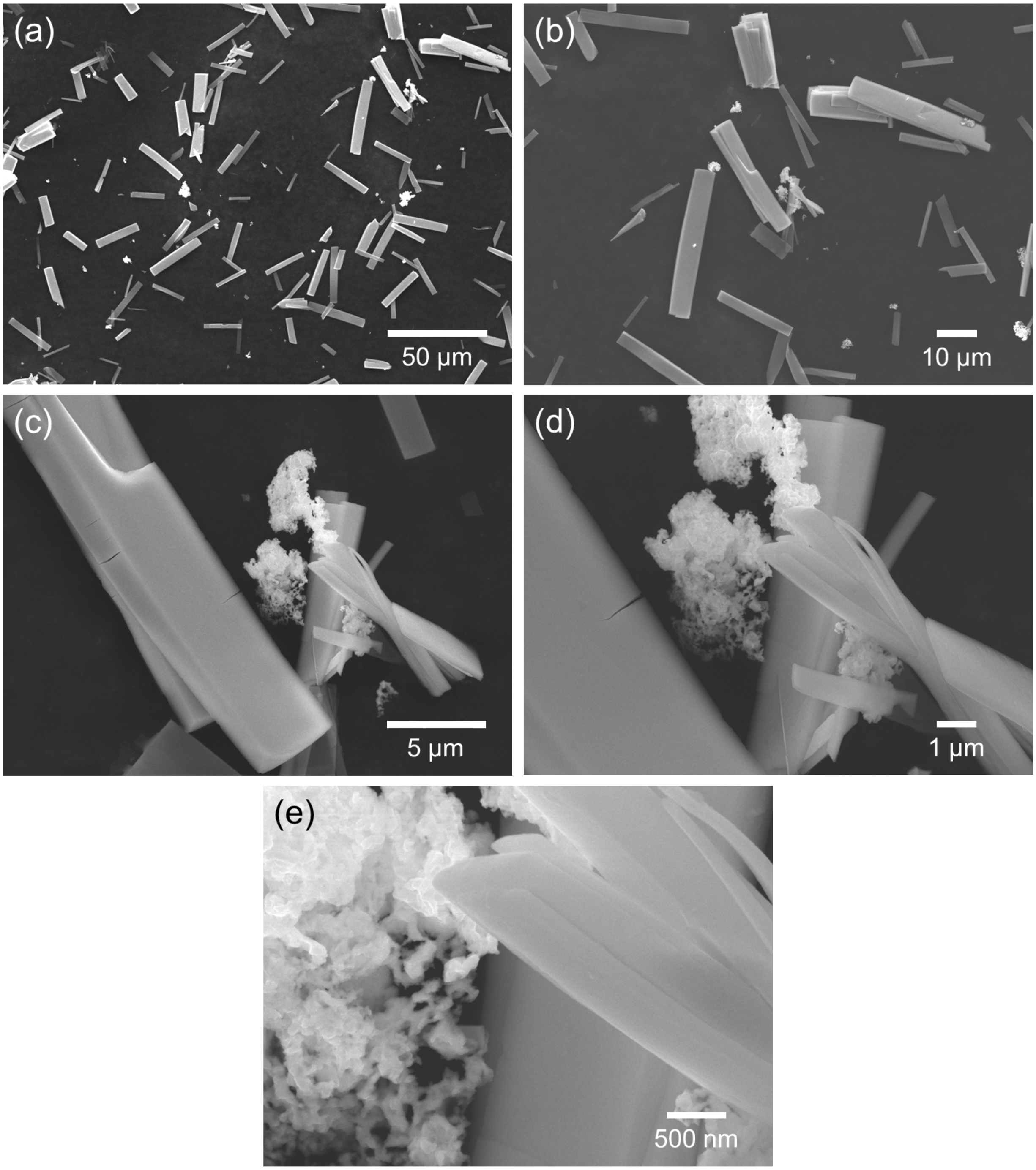

Figure S9. SEM images of replicated samples collected on day 1(freshly-made).

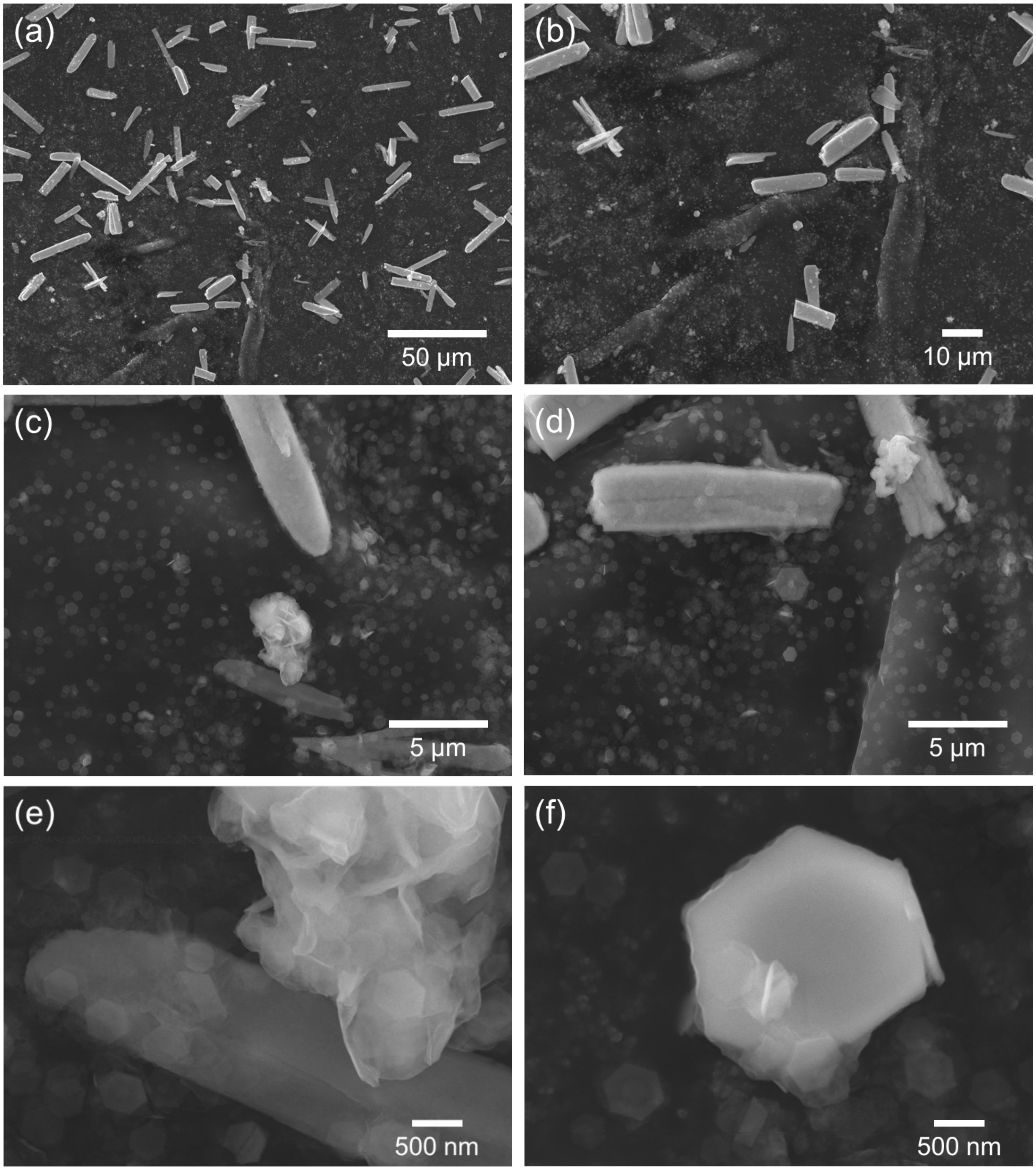

Figure S10. SEM images of replicated samples collected on day 4. (c) and (d) are from different locations of (a).

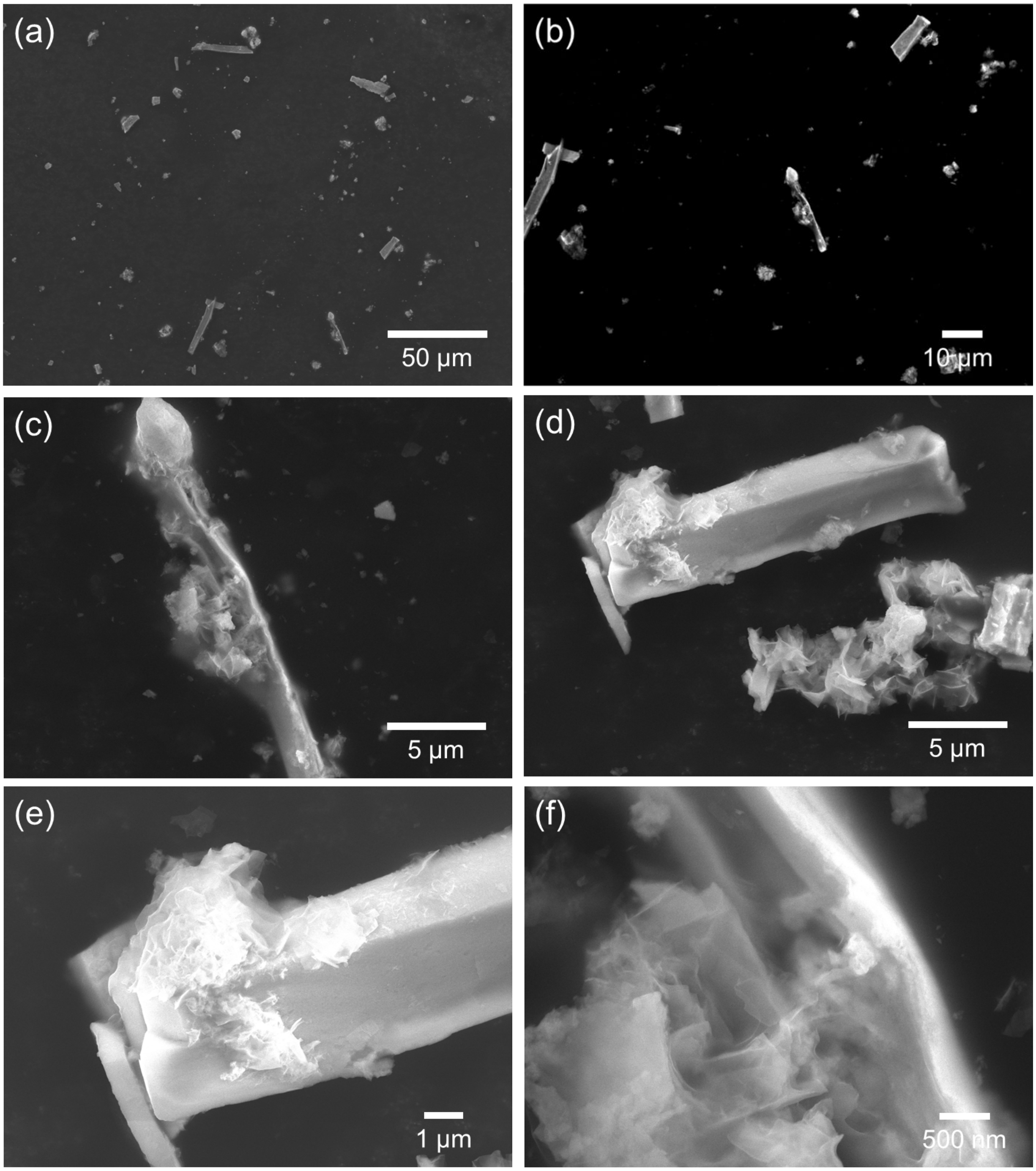

Figure S11. SEM images of replicated samples collected on day 7. (c) and (d) are from different locations of (a).

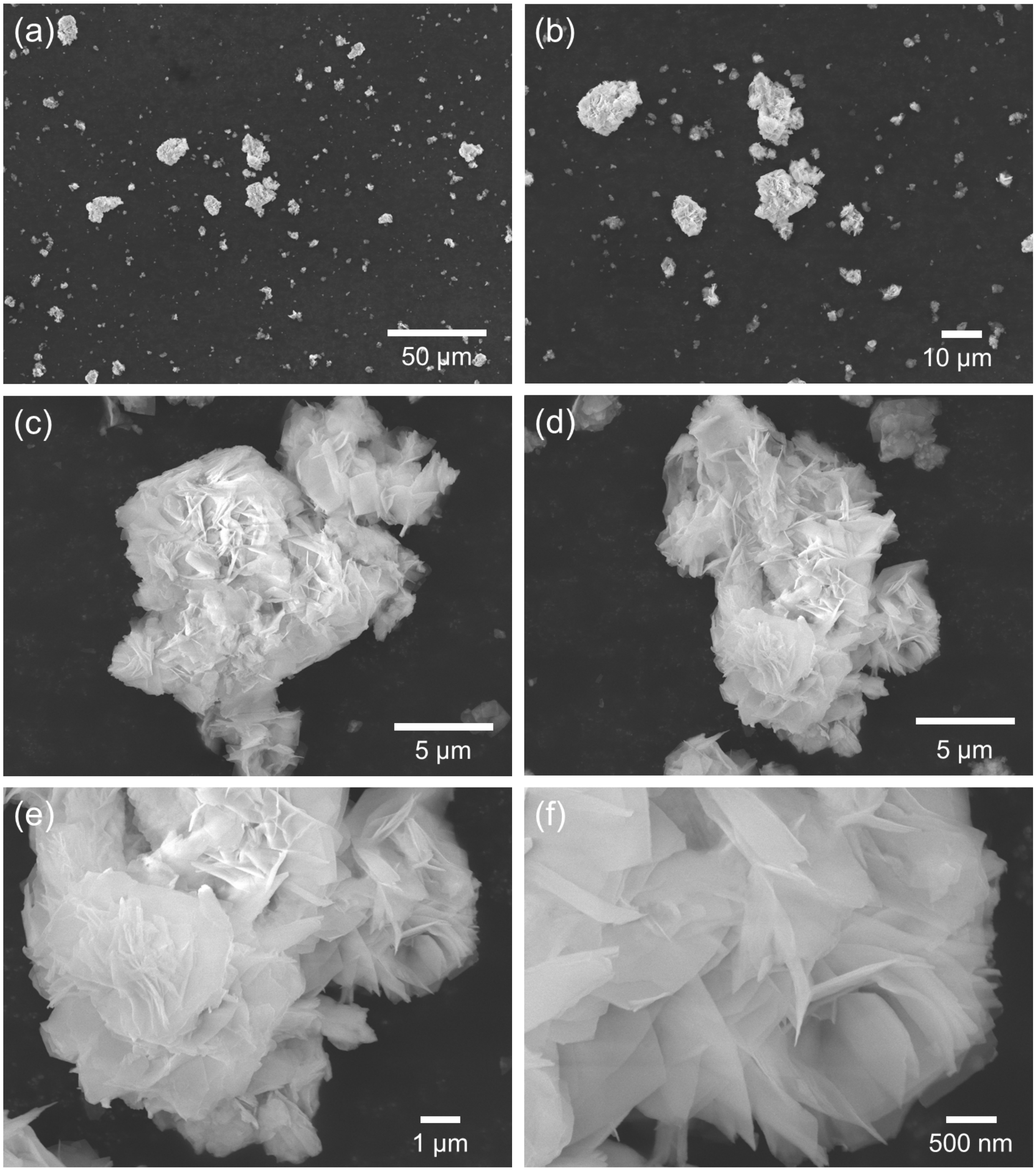

Figure S12. SEM images of replicated samples collected on day 10. (c) and (d) are from different locations of (a).

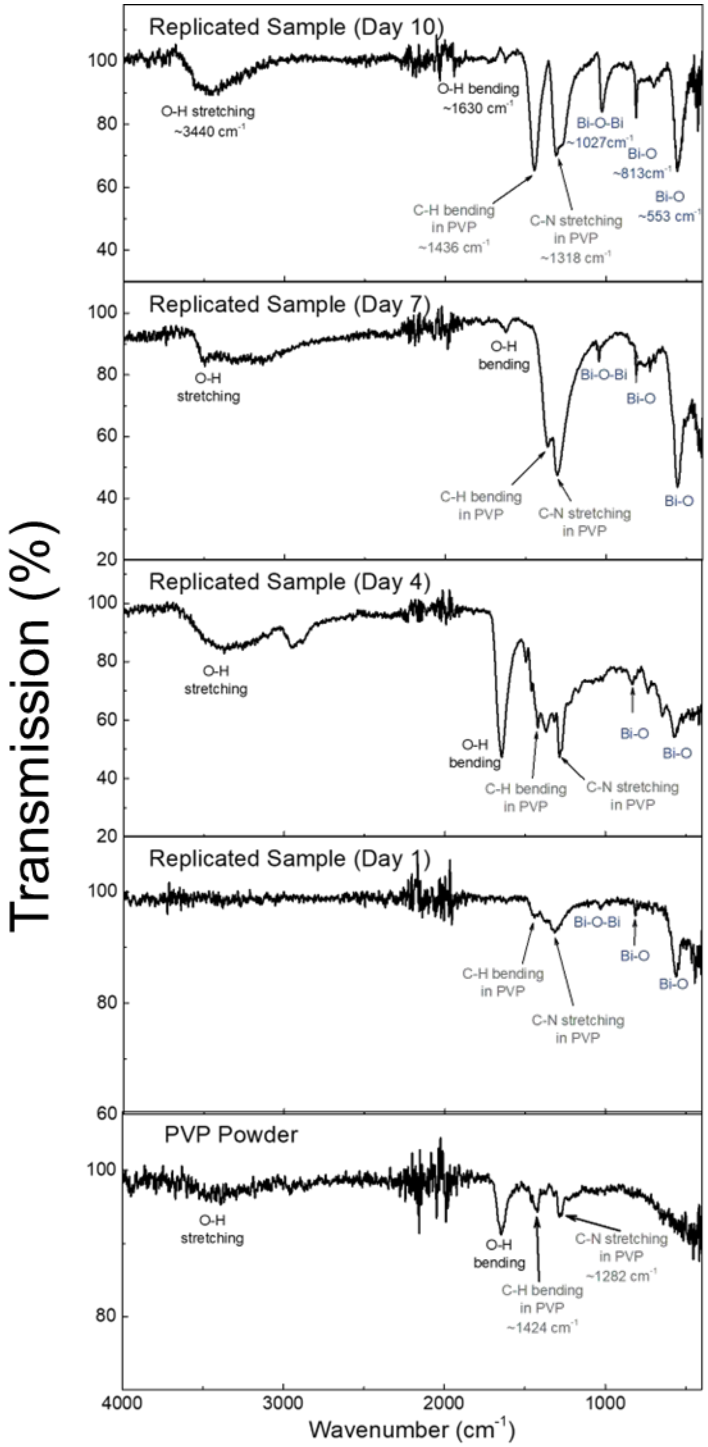

Figure S13. FTIR spectra of replicated samples collected on day 1(freshly-made), day 4, day 7 and day 10.
